# Depressive symptoms among people under COVID-19 quarantine or self-isolation in Korea: a propensity score matching analysis

**DOI:** 10.3389/fpsyt.2023.1255855

**Published:** 2023-12-18

**Authors:** Hyeon Sik Chu, Kounseok Lee

**Affiliations:** ^1^College of Nursing, Dankook University, Cheonan-si, Republic of Korea; ^2^Department of Psychiatry, College of Medicine, Hanyang University, Seoul, Republic of Korea

**Keywords:** COVID-19, quarantine, depression, anxiety, propensity scores, Korea

## Abstract

**Introduction:**

This study aims to determine the effect of COVID-19-related hospital isolation or self-isolation on depression using the propensity score matching method.

**Methods:**

Data on 217,734 participants were divided into groups based on whether or not they underwent quarantine for their COVID-19 diagnosis. COVID-19-related anxiety, depressive symptoms, subjective health status, and perceived stress were evaluated.

**Results:**

Based on the calculated propensity score, we matched the quarantined group and non-quarantined group using 1:2 matching with nearest neighbor matching and a caliper width of 0.1. Within the quarantined group, 16.4% of participants experienced significant depressive symptoms, which was significantly higher than that of the non-quarantined group. However, there was no significant difference between the two groups in COVID-19-related anxiety, self-rated health status, and perceived stress. In our multiple logistic regression analysis with related variables corrected, the quarantined group was 1.298 times more likely to have depressive symptoms than the non-quarantined group (95% CI = 1.030–1.634).

**Conclusion:**

Our study confirmed that COVID-19 quarantine is associated with depressive symptoms. These results indicate that healthcare policymakers and healthcare professionals must consider the negative mental and physical effects of quarantine when determining quarantine measures during an infectious disease disaster such as the COVID-19 pandemic.

## Introduction

The global public health crisis declared by the World Health Organization (WHO) on March 11, 2020, marked the onset of the novel coronavirus (COVID-19) pandemic (1). Korea’s COVID-19 prevention strategy, encapsulated in the 3 T approach (testing, tracing, and treating), involves widespread testing, the establishment of an advanced tracing system, and the implementation of rigorous treatment measures (2). In Korea, proactive measures are employed through innovative methods, including extensive testing to identify confirmed cases, tracing the infection paths of those cases, and isolating close contacts (3). However, the emergence of the delta variant was succeeded by the omicron variant, which exhibited double the infectivity of its predecessor, leading to a rapid increase in COVID-19 cases (4).

In contrast to natural disasters and accidents, infectious diseases present challenges with unclear risk factors, duration, and damage potential. These characteristics not only evoke collective fear, anxiety, and anger but also give rise to stigma and discrimination against various groups, including patients, their families, relevant workers, and residents of affected areas. Such stigma further escalates the risk of mental health issues (5, 6). Notably, isolation emerges as a significant risk factor for mental health problems associated with infectious diseases. Prior research indicates that individuals who undergo isolation are at least twice as likely to develop depressive disorders, anxiety disorders, and stress-related disorders compared to their non-isolated counterparts (7). The COVID-19 pandemic has exacerbated these challenges, with restricted international movement, paralyzed medical systems, and imposed lockdowns in multiple countries contributing to heightened stress and the pervasive fear of contracting the disease in people’s daily lives.

As the number of COVID-19 infections rises in Korea, there has been a heightened enforcement of vaccination and social distancing measures aimed at curbing the pandemic. Concurrently, stringent quarantine measures have been implemented for individuals infected with COVID-19 and their close contacts, either at home or in designated hospital facilities. Quarantine plays a crucial role in mitigating the risk of transmitting the virus by isolating and restricting the movement of individuals who may have been exposed to the infectious disease. In Korea, those who test positive for COVID-19 via PCR testing or upon arrival from abroad are mandated to undergo a 14-day quarantine and treatment period, either at home, in a designated facility, or at a hospital (2).

While these rigorous quarantine policies have proven effective in controlling the spread of COVID-19, they come with a range of adverse effects on individuals. Quarantine and isolation, integral to managing an infectious disease pandemic, introduce various psychological and environmental stressors, including concerns about infection, uncertainty regarding infectious diseases, and the disruption of social connections (8). Individuals afflicted with COVID-19 grapple with symptoms of the virus, shock, and isolation following an abrupt diagnosis, self-blame for infecting family members or close contacts, as well as experiences of exclusion and discrimination. Additionally, they may face significant disruptions in other facets of life, such as social and economic upheavals resulting from taking leaves of absence, job loss, or diminishing income (9–11).

Furthermore, individuals in contact with COVID-19 patients or those quarantined in facilities find themselves abruptly cut off from the outside world, compelled to endure isolated lives. Moreover, during epidemiological investigations, their personal information may be involuntarily exposed, leading to criticism, discrimination, and even ostracization.

Beyond the impact on mental health, depressive symptoms have been linked to various physical manifestations, including sleep disturbances, pain, and cognitive dysfunction in daily life. Consequently, these symptoms not only diminish the overall quality of life but also contribute to the emergence of suicidal thoughts and behaviors (12, 13).

Numerous studies conducted in multiple countries establish a connection between COVID-19-induced isolation and a spectrum of mental health issues such as depression, anxiety, post-traumatic stress disorder, and suicidal tendencies (14, 15). However, it is crucial to note that many of these studies have utilized a cross-sectional design without adequately controlling for confounding variables. Thus, there is a pressing need for longitudinal studies that can effectively control these variables and establish causality, shedding light on the specific effects of COVID-19-related quarantine on mental health (16).

One approach to addressing these challenges is to use propensity score matching (PSM) analysis in observational studies. PSM analysis calculates a propensity score that takes into account the influence of covariates between the experimental and control groups. By matching subjects, it assigns a randomly assigned effect, statistically correcting for selection bias and minimizing the effect of confounding variables (17).

This study aims to determine the effect of COVID-19-related hospital isolation or self-isolation on depression using the PSM method. Accordingly, we hypothesized that individuals undergoing COVID-19 quarantine or home quarantine would have higher levels of depressive symptoms than those who did not experience quarantine even after controlling other related factors such as anxiety and perceived stress.

In this way, efforts were made to quickly implement scientific preventive measures such as quarantine or home isolation in order to lessen the rate of mortality resulting from novel infections and to mitigate the spread of emerging infectious diseases in Korea. Research on the unfavorable effects of such preventive measures is still necessary, even with the impressive results. The foundational information gathered from these studies will help us respond to emerging infectious disease crises in the future with greater effectiveness.

## Methods

### Design

A secondary data analysis was conducted using a cross-sectional correlational study design with data obtained from the 2020 Korean Community Health Survey (KCHS).

### Setting and study participants

**Figure 1:**
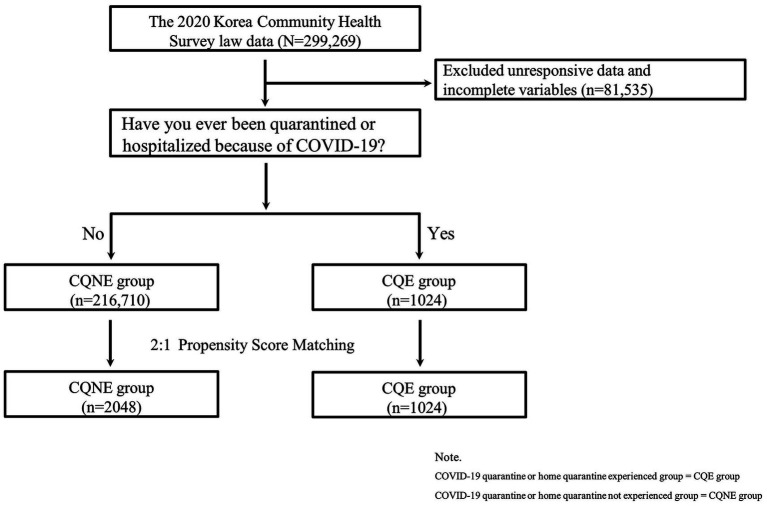
Flowchart of the study population.

We used data from the 2020 KCHS, a government-approved statistical survey by the Korea Disease Control and Prevention Agency. The KCHS is conducted annually per the Korean Community Health Act, and the target population is adults aged 19 years or older (18). The 2020 KCHS was conducted by community health centers across 17 metropolitan cities, covering 255 regional sites, from August 16 to October 31, 2020. In the 2020 KCHS, a trained interviewer directly visited the sample households and conducted face-to-face interviews using computer-assisted personal interviewing. Cases with missing values or incomplete variables (*n* = 81,535) were excluded from the dataset for this study. Overall data on 217,734 participants were included and analyzed (Figure 1).

### Measurements

#### Baseline characteristics of participants

Firstly, we defined baseline sociodemographic and health behavior-related variables. The social and demographic variables considered were age, gender, educational level, marital status, residence type, monthly average household income, employment status, and area of residence. The health behavior-related variables consisted of obesity, smoking, binge drinking, diabetes, and high blood pressure. Education level was classified as elementary school graduation or less, middle school graduation or less, high school graduation or less, college graduation or less, and university graduation or less. Residence type was classified as either living with others or living alone. Monthly household income was classified as quartiles. Employment status was classified as having a job or not. Residential area was classified as urban or rural. Based on body mass index (BMI), obesity was classified as underweight (<18.5), normal (≥18.5 and ≤ 24.9), obese (≥25.0 and ≤ 29.9), or highly obese (≥30). Smoking was classified as non-smoking, past smoking, or smoking. Binge drinking was defined as consuming more than seven drinks during a single occasion at least once a month. Finally, hypertension was defined as being diagnosed with hypertension by a doctor and being prescribed anti-hypertensive drugs.

#### Definition of a person who experienced COVID-19 quarantine

Individuals who answered “yes” to the question “Have you been quarantined or hospitalized for COVID-19 since January 2020?” in the 2020 KCHS were defined as persons who experienced COVID-19 quarantine.

#### Subjective health status and perceived stress

Subjective health status was based on the response to “How do you usually feel about your health?” The answers “very poor” and “bad” were reclassified as poor, “average” was reclassified as moderate, and “very good” and “good” were reclassified as good. As for perceived stress, participants answered “How much stress do you usually feel in your daily life?” The responses “never” and “sometimes” were considered low, while “fairly often” and “very often” were considered high.

#### COVID-19-related anxiety

COVID-19-related anxiety was the focus of the following 2020 KCHS question: “How worried are you about the following statements in light of the COVID-19 outbreak?” The statements were as follows: “I am concerned that I will die if I become infected with COVID-19,” “I am concerned that if I become infected, I will be criticized or ostracized by those around me,” and “I am concerned that the COVID-19 pandemic will cause economic damage to me and my family.” Each statement was ranked on a five-point Likert scale, with one denoting “not at all” and five denoting “strongly agree.” In this study, the score range for COVID-19-related anxiety was 3–5; moreover, higher total sums represented higher levels of anxiety related to COVID-19. The reliability of this tool was measured using Cronbach’s α, which was 0.64.

#### Depressive symptoms

Depressive symptoms were measured using the Korean version of the Patient Health Questionnaire-9 (PHQ-9). The PHQ-9 consists of nine symptoms that correspond with the diagnostic criteria for major depression in the Diagnostic and Statistical Manual of Mental Disorders, fourth edition (DSM-IV). It asks how often the patient has experienced certain symptoms in the preceding 2 weeks (19). Responses are evaluated on a four-point Likert scale that includes “never,” “for a few days,” “more than a week,” and “almost every day”; moreover, the total score ranges from 0 to 27. For this test, the cut-off point to be defined as a clinically significant depressive symptom was set to 10, and Cronbach’s α was 0.81 (20).

### Data analysis

All statistical analyses were performed using SPSS 23.0 and R.^2^ To reduce selection bias by confounding covariates, PSM was performed using R. In PSM, the propensity score represents the probability that a study subject will be included in the treatment group (or vice versa) rather than in the control group (21). Based on the calculated propensity score, we matched the quarantined group and non-quarantined group using 1:2 matching with nearest neighbor matching and a caliper width of 0.1.

**Figure 2:**
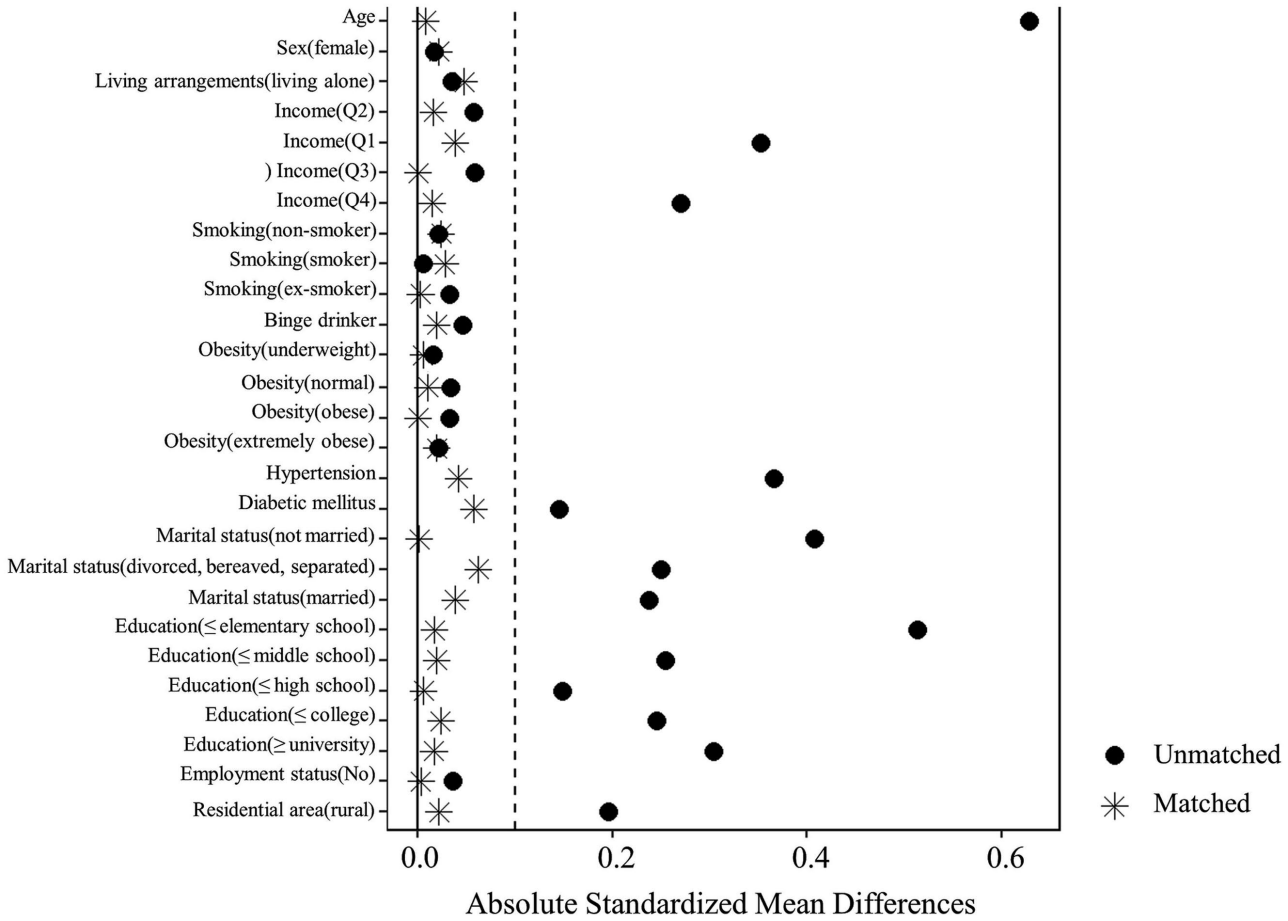
Absolute standardized differences of the 13 variables for propensity score matching analysis.

Participants from each group were matched based on the 13 baseline characteristics (age, gender, education level, marital status, living arrangements, monthly household income, employment status, residential area, obesity, smoking, binge drinking, diabetes, and hypertension), and an estimated logit width of 0.1 standardized difference was used. For each propensity model covariate, the absolute standardized difference was calculated before and after as less than 10%, which implies a well-controlled balance between the two groups. Figure 2 shows the absolute standardized difference of the 13 matched and unmatched variables for PSM analysis.

For continuous and categorical variables, an independent *t*-test and χ^2^ test, respectively, were performed. Multiple logistic regression analysis was also conducted to determine predictive factors. The enter technique was applied for variable entry in the logistic regression analysis, with perceived stress, subjective health status, and COVID-19-related anxiety included as covariates. A two-tailed *p* < 0.05 was considered statistically significant.

## Results

### Participant characteristics from unadjusted data

The sociodemographic and health behavior characteristics of participants who experienced COVID-19 quarantine in the hospital or at home are summarized in Table 1. A total of 1,024 subjects experienced COVID-19 quarantine or self-isolation among the participants in this study.

**Table 1:** Comparison of baseline characteristics between the two groups before and after propensity score matching (*N* = 3,072).

Variable	Categories	Unadjusted data			Propensity score-matched data		
		CQNE group	CQE group	*p* value	CQNE group	CQE group	*p* value
		(*n* = 216,710)	(*n* = 1,024)		(*n* = 2,048)	(*n* = 1,024)	
		*n* (%) or M ± SD	*n* (%) or M ± SD		*n* (%) or M ± SD	*n* (%) or M ± SD	
Age		54.15 ± 17.55	43.42 ± 17.09	<0.001	43.28 ± 16.94	43.42 ± 17.09	0.846
Sex	Male	99,761 (46.0%)	480 (46.9%)	0.612	982(47.9)	480 (46.9%)	0.601
	Female	116,949 (54.0%)	544 (53.1%)		1,066(52.1)	544 (53.1%)	
Educational attainments	≤ Elementary school	45,227 (20.9%)	76 (7.4%)	<0.001	143 (7.0%)	76 (7.4%)	0.938
	≤ Middle school	24,713 (11.4%)	57 (5.6%)		123 (6.0%)	57 (5.6%)	
	≤ High school	64,140 (29.6%)	239 (23.3%)		473 (23.1%)	239 (23.3%)	
	≤ College	34,969 (16.1%)	277 (27.1%)		575 (28.1%)	277 (27.1%)	
	≥ University	47,661 (22.0%)	375 (36.6%)		734 (35.8%)	375 (36.6%)	
Marital status	Not married	38,319 (17.7%)	383 (37.4%)	<0.001	767 (37.5%)	3,527 (51.5%)	0.21
	Married	137,226 (63.3%)	527 (51.5%)		1,093 (53.4%)	527 (51.5%)	
	Divorced/bereaved/separated	41,165 (19.0%)	114 (11.1%)		188 (9.2%)	114 (11.1%)	
Living arrangements	Living with others	182,944 (84.4%)	877 (85.6%)	0.3	1,788 (87.3%)	877 (85.6%)	0.221
	Living alone	33,766 (15.6%)	147 (14.4%)		260 (12.7%)	147 (14.4%)	
Monthly household income	Q1	60,468 (27.9%)	156 (15.2%)	<0.001	284 (13.9%)	156 (15.2%)	0.773
	Q2	59,989 (27.7%)	258 (25.2%)		530 (25.9%)	258 (25.2%)	
	Q3	55,861 (25.8%)	291 (28.4%)		582 (28.4%)	291 (28.4%)	
	Q4	40,392 (18.6%)	319 (31.2%)		652 (31.8%)	319 (31.2%)	
Employment status	No	83,798 (38.7%)	378 (36.9%)	0.264	759 (37.1%)	378 (36.9%)	0.968
	Yes	132,912 (61.3%)	646 (63.1%)		1,289 (62.9%)	646 (63.1%)	
Residential area	Urban	122,943 (56.7%)	676 (66.0%)	<0.001	1,331 (65.0%)	676 (66.0%)	0.601
	Rural	93,767 (43.3%)	348 (34.0%)		717 (35.0%)	348 (34.0%)	
Obesity	Underweight	8,896 (4.1%)	39 (3.8%)	0.59	76 (3.7%)	39 (3.8%)	0.953
	Normal	141,640 (65.4%)	653 (63.8%)		1,316 (64.3%)	653 (63.8%)	
	Obese	57,810 (26.7%)	288 (28.1%)		576 (28.1%)	288 (28.1%)	
	Extremely obese	8,364 (3.9%)	44 (4.3%)		80 (3.9%)	44 (4.3%)	
Smoking	Non-smoker	140,652 (64.9%)	675 (65.9%)	0.602	1,373 (67.0%)	675 (65.9%)	0.749
	Ex-smoker	40,112 (18.5%)	177 (17.3%)		352 (17.2%)	177 (17.3%)	
	Smoker	35,946 (16.6%)	172 (16.8%)		323 (15.8%)	172 (16.8%)	
Binge drinker	No	187,513 (86.5%)	869 (84.9%)	0.131	1,752 (85.5%)	869 (84.9%)	0.652
	Yes	29,197 (13.5%)	155 (15.1%)		296 (14.5%)	155 (15.1%)	
Diabetic mellitus	No	193,432 (89.3%)	952 (93.0%)	<0.001	1,934 (94.4%)	952 (93.0%)	0.127
	Yes	23,278 (10.7%)	72 (7.0%)		114 (5.6%)	72 (7.0%)	
Hypertension	No	159,863 (73.8%)	884 (86.3%)	<0.001	1,797 (87.7%)	884 (86.3%)	0.292
	Yes	56,847 (26.2%)	140 (13.7%)		251 (12.3%)	140 (13.7%)	

CQE, COVID-19 quarantine or home quarantine experienced; CQNE, COVID-19 quarantine or home quarantine not experienced.

The quarantined group had a lower age, higher education level, and more unmarried people than that of the non-quarantined group. Among all participants, the income level was high, the residential area was mainly urban, and the prevalence of diabetes and hypertension was low. However, there was no significant difference in all variables between the two groups after 1:2 propensity score matching.

### Comparison of health status between the two groups after propensity score matching

Table 2 compares the health status of the quarantined group and the non-quarantined group after 1:2 propensity score matching. There was a statistically significant difference between the two groups in depressive symptoms based on the presence or absence of COVID-19-related isolation (χ^2^ = 4.098, *p* = 0.045). While 16.4% of the quarantined group experienced a significant rate of depressive symptoms, 13.7% of the non-quarantined group had a significantly lower rate. However, there was no statistically significant difference between the groups in COVID-19-related anxiety, subjective health status, and perceived stress.

**Table 2:** Matched comparison of health status between CQNE group and CQE group (*N* = 3,072).

Variables	Categories	CQNE group (*n* = 2,048)	CQE group (*n* = 1,024)	χ^2^ or *t*	*p* value
		*n* (%) or M ± SD	*n* (%) or M ± SD		
Depressive symptoms (PHQ-9)	Non-depressed	1,768 (86.3)	856 (83.6)	4.098	0.045
	Depressed	280 (13.7)	168 (16.4)		
COVID-19 related anxiety		11.06 ± 2.49	11.03 ± 2.62	0.348	0.728
Subjective health status	Good	1,215 (59.3)	77 (7.5)	1.940	0.382
	Moderate	696 (34.0)	325 (31.7)		
	Poor	137 (6.7)	622 (60.8)		
Perceived stress	Low	1,506 (73.5)	760 (74.2)	0.165	0.696
	High	542 (26.5)	264 (25.8)		

CQE, COVID-19 quarantine or home quarantine experienced; CQNE, COVID-19 quarantine or home quarantine not experienced.

### Effect of COVID-19 quarantine on depressive symptoms

**Figure 3:**
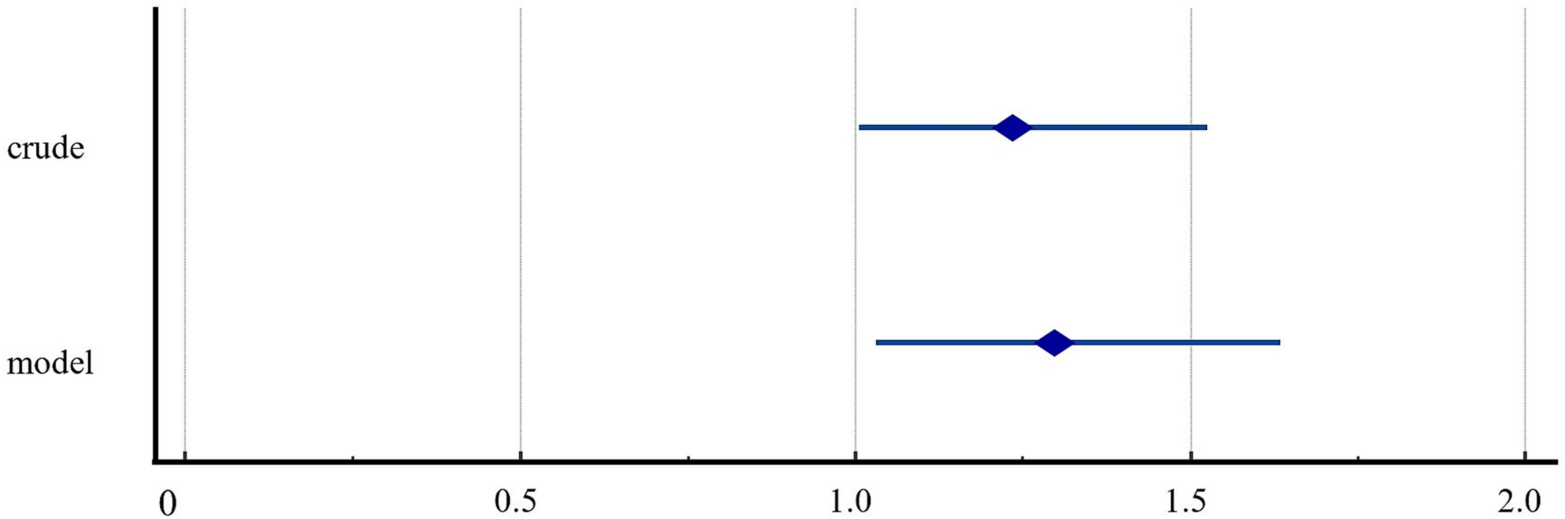
Forest plot on the risk of depression in individuals who experienced COVID-19 quarantine.

Table 3 and Figure 3 summarize the results of univariate and multivariate logistic regression analyses. The results of all univariate logistic regression models were statistically significant. Thus, the probability of having significant depressive symptoms was 1.239 times higher in the quarantined group than in the non-quarantined group (95% CI = 1.007–1.526).

**Table 3:** The effect of COVID-19 quarantine on depressive symptoms (*N* = 3,072).

	Propensity-matched data			
	Crude		Model†	
	OR (95% CI)	*p* value	OR (95% CI)	*p* value
Person who COVID-19 quarantine not experienced (*n* = 2,048)	1		1	
Person who COVID-19 quarantine experienced (*n* = 1,024)	1.239 (1.007–1.526)	0.043	1.298 (1.030–1.634)	0.027

†Adjusted for COVID-19 related anxiety, subjective health status, and perceived stress; Cox and Snell *R*^2^ = 0.141; Nagelkerke *R*^2^ = 0.249; OR, Odds ratio; CI, Confidence interval.

Multivariate logistic regression analysis, adjusted for subjective health status, COVID-19-related anxiety, and perceived stress, revealed that the probability of having a significant depressive symptom was 1.298 times higher in the quarantined group than in the non-quarantined group (95% CI = 1.030–1.634). This particular model’s Cox and Snell *R*^2^ values and Nagelkerke *R*^2^ values were 0.141 and 0.249, respectively.

## Discussion

This study confirmed the relationship between COVID-19-related isolation and depressive symptoms. Even after controlling for sociodemographic and other health related variables, experiencing COVID-19-related isolation significantly increased the depressive symptoms.

During an infectious disease outbreak, various stressors may arise depending on the characteristics of the disease and quarantine policies. To prevent the spread of infection, many studies recommend maintaining good hygiene and utilizing social distancing (22). Depression may result from this isolation process; however, if a person has pre-existing depressive symptoms, such isolation may affect emotions even in the undiagnosed sub-syndrome group. Mental health challenges that quarantined persons may experience include anxiety, anger, feelings of isolation, boredom, insomnia, and suicidal thoughts (23).

After controlling for sociodemographic variables using the PSM in this study, the proportions of those with significant depressive symptoms in the COVID-19 quarantine or home quarantine experienced group and the non-experienced group were 16.4 and 13.3%, respectively. In a Chinese study of the general population, 28.8 and 16.5% of people complained of moderate-to-severe anxiety and depression, respectively (24). In Italy, reports showed mild post-traumatic stress symptoms in 37.1% of the population and severe symptoms in 20.8 and 17.3% (25). In a cross-sectional study of confirmed COVID-19 patients, 97.2% of patients experienced depressive symptoms and anxiety (26). In studies conducted in Argentina, the findings indicate that isolation poses a risk factor for mental health, particularly for women, young individuals, and those with a history of mental illness (15, 27). Another study has found a decrease in depressive symptoms with increased mobility (28). The significant difference in rates of depressive symptoms between the present study and previous studies may be because we controlled for sociodemographic variables to compare differences between groups within the present study. In addition, it may be related to the modalities of the COVID-19 epidemic across countries and mortality rates from COVID-19, which may influence individuals’ perceived severity of illness (29).

In this study, we aimed to examine the psychological vulnerability to depression in individuals who experienced COVID-19-related quarantine by comparing the differences between those who experienced quarantine and those who did not, using empirical data. We found that after controlling for sociodemographic variables, there were no significant differences between the two groups in COVID-19-related anxiety, subjective health, and perceived stress levels, except for the mental health variable depressive symptoms, contrary to our hypothesis. However, previous studies have reported that COVID-19-related quarantine not only increases depression and anxiety, but also exacerbates pre-existing mental illness in individuals (30, 31). Furthermore, individuals who experienced quarantine due to COVID-19 infection had significantly higher stress levels (32). The findings of this study may be inconsistent with those of previous studies because we controlled for sociodemographic variables that may affect mental health with PSM, which did not show a difference between the two groups. In addition, it may be that during the COVID-19 pandemic, not only the quarantine-experienced group but also the non-quarantine-experienced group experienced social isolation due to various prevention policies, including social distancing, related to COVID-19 infection (33).

In the present study, depressive symptoms were significantly higher in the quarantined group; conversely, there was no difference in anxiety level between the groups. In a previous study, among COVID-19 patients who were hospitalized, 50% experienced depressive symptoms during hospital isolation, but this decreased to 10% after discharge (9). There were also significant symptoms of anxiety during treatment, which also decreased after discharge. However, understanding that anxiety is considerably influenced by situational aspects, we could not confirm these results because this study did not compare anxiety before and after isolation.

The emotional difficulties associated with COVID-19 may be more likely to appear if they are accompanied by a history of psychiatric illness, social stigma, unstable employment status, and a long quarantine period (26, 34). A study of hospitalized COVID-19 patients reported that they received more antipsychotics and benzodiazepines than patients who did not have COVID-19 (35).

Patients with a psychiatric history experienced an exacerbation of their psychiatric issues during the COVID-19 pandemic. Additionally, a report revealed that initial psychosis, delirium, and mood disorders may have appeared for the first time in patients without pre-existing psychiatric diseases (36). The results of this study showed that the isolated group had significantly higher depressive symptoms. Though these results did not confirm the causal relationship between depressive symptoms and isolation, it is highly likely that isolation increased the depressive symptoms.

Furthermore, the characteristics of COVID-19 itself include the ability to infect others (transmissibility), the fact that it is a new infectious disease (lack of information), and the uncertainty of its future trends (unpredictability). Indeed, an epidemic is not a one-time event but a series of occurrences until it is resolved. An infectious disease patient is not only a victim of a disaster but also a perpetrator who can transmit the disease (37); therefore, they are often criticized, shunned, and may experience guilt for having infected or quarantined family or friends (9). For some patients, experiencing the death of a family member in isolation may impede the natural mourning process (38).

As previously mentioned, among 10 patients who were hospitalized with mild COVID-19 pneumonia, 50% had depressive symptoms during treatment, but this decreased to 10% after discharge (9). By contrast, in a study of 107 patients who had no symptoms or very mild symptoms, 24% complained of depression, 15% complained of anxiety, and 11.2% complained of suicidal thoughts during the first week of admission (34). This is consistent with the results of this study, i.e., that isolation or hospitalization itself significantly affects depressive symptoms.

However, the opposite scenario has also been observed. In a Chinese study conducted in February 2020, approximately 20% of 50 people quarantined for COVID-19 experienced anxiety and depression; incidentally, the percentage was not significantly different from that of the control group who did not undergo quarantine (39). In a Korean study, the rate of post-traumatic stress symptoms among quarantined individuals was higher than that of the general population (25 and 10%, respectively) (40). However, follow-up studies are required to confirm this, given the small sample size of quarantined persons.

In any case, support for maintaining and promoting mental health is needed for COVID-19 patients who are quarantined in hospitals and for close contacts who are self-isolating at home (41). To reduce the loneliness caused by isolation, remote communication using smartphones must be encouraged; additionally, isolated individuals who complain of depression and anxiety need interventions such as evaluation and counseling by a mental health professional using remote communication (42, 43). In addition, providing accurate and prompt information related to COVID-19 is necessary to reduce uncertainty in those infected with COVID-19 and those in self-quarantine. Policy support that can minimize the effect of social stigma related to contracting COVID-19 is also needed.

This study is a secondary analysis using data from the 2020 KCHS. The primary survey did not classify respondents based on those who experienced hospitalization due to COVID-19 and those who self-isolated to prevent the spread of COVID-19. As a result, the effect of COVID-19 itself on depressive symptoms could not be controlled. In addition, the amount of time between the quarantine or self-isolation and the survey date was not known. Therefore, the results reflect the participants’ status immediately after the impact of the infectious disease, and additional research is needed to confirm the long-term effects. Certain other potential factors were also excluded. Furthermore, in interpreting the results, it is crucial to consider that the main variables were measured using self-report questionnaires, making it impossible to control biases such as social desirability or recall bias. Because of the cross-sectional study design, it is not possible to draw firm conclusions on the association between depressive symptoms and COVID-19-related isolation. And this study was conducted in one country and there may be limitations in generalizing the findings to other cultural contexts or regions with different healthcare systems and social settings. Therefore, it is crucial to conduct longitudinal studies in various countries to investigate the impact of quarantine, related to COVID-19, on mental health.

Nevertheless, this study evaluated the COVID-19 related anxiety, depressive symptoms, and stress experienced by participants, inclusive of their quarantine status. In addition, the effect of confounding variables was statistically corrected by matching the propensity score to improve the balance between groups. This study confirmed that the probability of experiencing depressive symptoms was relatively high in the group that experienced COVID-19-related isolation.

## Conclusion

In short, this study attempted to confirm the effect of COVID-19-related quarantine on depression by using the PSM method. The probability of depressive symptoms was significantly higher in the quarantined group than in the non-quarantined group. The results of this study reveal the need for mental health resources and support for people undergoing COVID-19-related quarantine. When coordinating the appropriate support based on the characteristics of a disaster, such as an infectious disease pandemic, it is necessary to consider mental health issues as well as physical health.

## Data availability statement

The original contributions presented in the study are included in the article/supplementary material, further inquiries can be directed to the corresponding author.

## Ethics statement

The study procedures were reviewed and approved by the Hanyang University Institutional Review Board (IRB No. HYUIRB-202201-012). Written informed consent was not required in accordance with local and national guidelines.

## Author contributions

HC: Conceptualization, Data curation, Visualization, Formal Analysis, Methodology, Writing – original draft. KL: Conceptualization, Data curation, Visualization, Funding acquisition, Resources, Supervision, Validation, Writing – review & editing.

## Footnotes

^2^ https://www.r-project.org
